# Predictive validity of the ACS-NSQIP surgical risk calculator in geriatric patients undergoing lumbar surgery

**DOI:** 10.1097/MD.0000000000008416

**Published:** 2017-10-27

**Authors:** Xiao Wang, Yanting Hu, Binjiang Zhao, Yue Su

**Affiliations:** a Beijing Shijitan Hospital; b Anesthesiology, Capital Medical University, Beijing, China.

**Keywords:** ACS-NSQIP, lumbar spinal surgery, postoperative complications, senior patients

## Abstract

The risk calculator of the American College of Surgeons National Surgical Quality Improvement Program (ACS-NSQIP) has been shown to be useful in predicting postoperative complications. In this study, we aimed to evaluate the predictive value of the ACS-NSQIP calculator in geriatric patients undergoing lumbar surgery.

A total of 242 geriatric patients who underwent lumbar surgery between January 2014 and December 2016 were included. Preoperative clinical information was retrospectively reviewed and entered into the ACS-NSQIP calculator. The predictive value of the ACS-NSQIP model was assessed using the Hosmer–Lemeshow test, Brier score (B), and receiver operating characteristics (ROC, also referred C-statistic) curve analysis. Additional risk factors were calculated as surgeon-adjusted risk including previous cardiac event and cerebrovascular disease.

Preoperative risk factors including age (*P* = .004), functional independence (*P* = 0), American Society of Anesthesiologists class (ASA class, *P* = 0), dyspnea (*P* = 0), dialysis (*P* = .049), previous cardiac event (*P* = .001), and history of cerebrovascular disease (*P* = 0) were significantly associated with a greater incidence of postoperative complications. Observed and predicted incidence of postoperative complications was 43.8% and 13.7% (±5.9%) (*P* *<* .01), respectively. The Hosmer–Lemeshow test demonstrated adequate predictive accuracy of the ACS-NSQIP model for all complications. However, Brier score showed that the ACS-NSQIP model could not accurately predict risk of all (B = 0.321) or serious (B = 0.241) complications, although it accurately predicted the risk of death (B = 0.0072); this was supported by ROC curve analysis. The ROC curve also showed that the model had high sensitivity and specificity for predicting renal failure and readmission.

The ACS-NSQIP surgical risk calculator is not an accurate tool for the prediction of postoperative complications in geriatric Chinese patients undergoing lumbar surgery.

## 1 Introduction

Lumbar spinal stenosis frequently affects the mobility and quality of life for millions of people, especially the geriatric population. Worldwide, there has been an increase in the need for lumbar surgery in tandem with the phenomenon of population aging.^[1]^ Higher age is a proven significant risk factor for surgical complications.^[2,3]^ Therefore, accurate prediction of the risk of complications in elderly patients undergoing lumbar surgery will help reduce the incidence of postoperative complications. In 2013, The American College of Surgeons National Surgical Quality Improvement Program (ACS-NSQIP)^[4]^ developed an online surgical risk calculator, based on analysis of a database of preoperative information and postoperative complications pertaining to more than a million surgical patients across 393 hospitals in the United States.^[5]^ The surgical risk calculator found widespread application in the clinical setting in over 1500 types of surgeries,^[5,6]^ although with some exceptions including lumbar surgery. This tool helps estimate the risk of postoperative complications based on preoperative data such as patient age, body mass index (BMI), and comorbid conditions (eg, diabetes and hypertension). This study was conducted to evaluate the predictive utility of the ACS-NSQIP surgical risk calculator in geriatric patients undergoing lumbar surgery.

## 2 Material and methods

Elderly patients (age > 60 years) with isolated spinal stenosis who underwent conventional laminectomy without fusion in the period between January 2014 and December 2016 at the Beijing Shijitan Hospital, China were screened for inclusion in the study. Conventional laminectomy was performed in these patients through a midline incision, and the spinous process and lamina removed. Extirpation of herniated discs was performed only in cases where disc bulging or herniation contributed to stenosis. Table 1 summarizes the selection criteria for the inclusion of patients in the study. A total of 242 patients were included in the final analysis in this retrospective study. The study was approved by the institutional ethics committee, and informed consent was obtained from all individual subjects or their guardians.

**Table 1:**
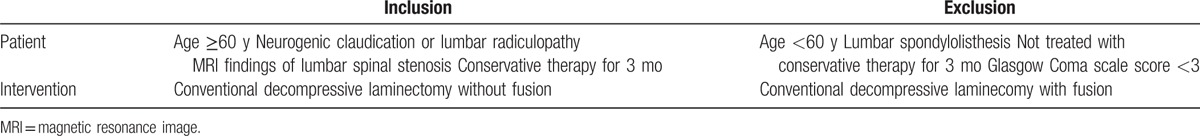
Summary of inclusion and exclusion criteria.

Patient information was retrospectively obtained from hospital medical records or telephonic interviews of the patients or their guardians. Demographic data included age, gender, smoking habits, weight, height, and functional status. Clinical information was collected according to the requirements of the ACS-NSQIP risk calculator and comprised preoperative assessments for American Society of Anesthesiologists class, diabetes, hypertension, congestive heart failure, dyspnea, ascites, chronic obstructive pulmonary disease, dialysis, renal failure, sepsis, ventilator dependence, and disseminated cancer. Further, surgical parameters, including emergent or elective operation and current procedural terminology code (22612; arthrodesis, posterior or posterolateral technique, single-level, lumbar, with or without lateral transverse technique), were recorded.

### 2.1 Risk calculation

**Figure 1:**
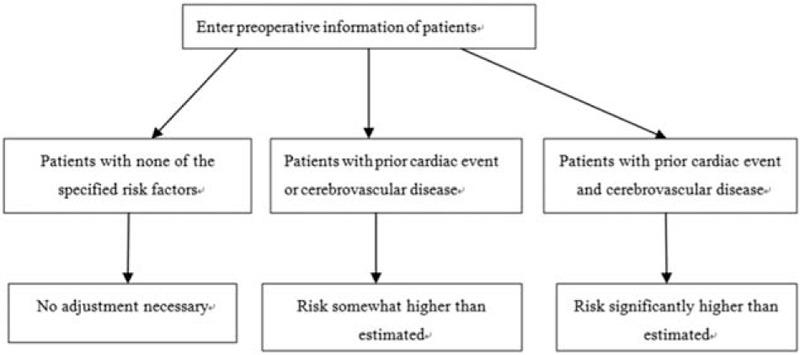
Flow chart of risk-adjusted subgroups.

Data were further categorized as indicated by the ACS-NSQIP surgical risk calculator. Based on the BMI (kg/m^2^), patients were further classified into 6 groups: low weight (BMI <18.5); normal weight (≥18.5 to ≤25); overweight (>25 to ≤30); obesity level I (>30 to ≤35); obesity level II (>35 to ≤40); and obesity level III (BMI >40). Further, 3 risk-adjusted subgroups, based on surgeon's assessment of risk, were included: “no adjustment necessary”, “risk somewhat higher than estimated”, and “risk significantly higher than estimated”, on the basis of additional risk factors including prior cardiac event and cerebrovascular disease. Patients with none, 1, or more than 2 of the specified risk factors were assigned to the “no adjustment necessary”, “risk somewhat higher than estimated”, and “risk significantly higher than estimated” categories, respectively (Fig. 1).

### 2.2 Statistical analysis

All data were analyzed using SPSS v. 22.0 (IBM SPSS Statistics, NY). Quantitative data are presented as mean ± standard deviation, and categorical data as frequencies and percentages. Chi-square test was used to assess the difference between the overall predicted risk and the observed risk of complications. Wilcoxon signed-rank test was used to compare hospital stay between study groups. The Hosmer–Lemeshow goodness-of-fit test and receiver-operating characteristics (also referred C-statistic) curve analysis were performed to evaluate the predictive validity of the ACS-NSQIP surgical risk calculator. Brier score (B) was used to assess the deviation between the predicted and observed outcomes, and a score <0.01 indicated predictive precision >90%.^[5,7,8]^

## 3 Results

### 3.1 Patient characteristics

All 242 subjects (mean age: 78.5 ± 7.2 years, range 60–93 years) underwent conventional laminectomy without fusion (mean operative time: 6.5 ± 2.1 hours). Baseline patient characteristics and preoperative parameters, based on the ACS-NSQIP risk calculator, are presented in Table 2. Postoperative complications were significantly associated with age (*P* = .004), functional independence (*P* = 0), American Society of Anesthesiologists class (*P* = 0), dyspnea (*P* = 0), dialysis (*P* = .049), previous cardiac event (*P* = .001), and history of cerebrovascular disease (*P* = 0). However, other variables captured for risk assessment were not associated with postoperative complications (*P* > .05 for all; Table 2).

**Table 2:**
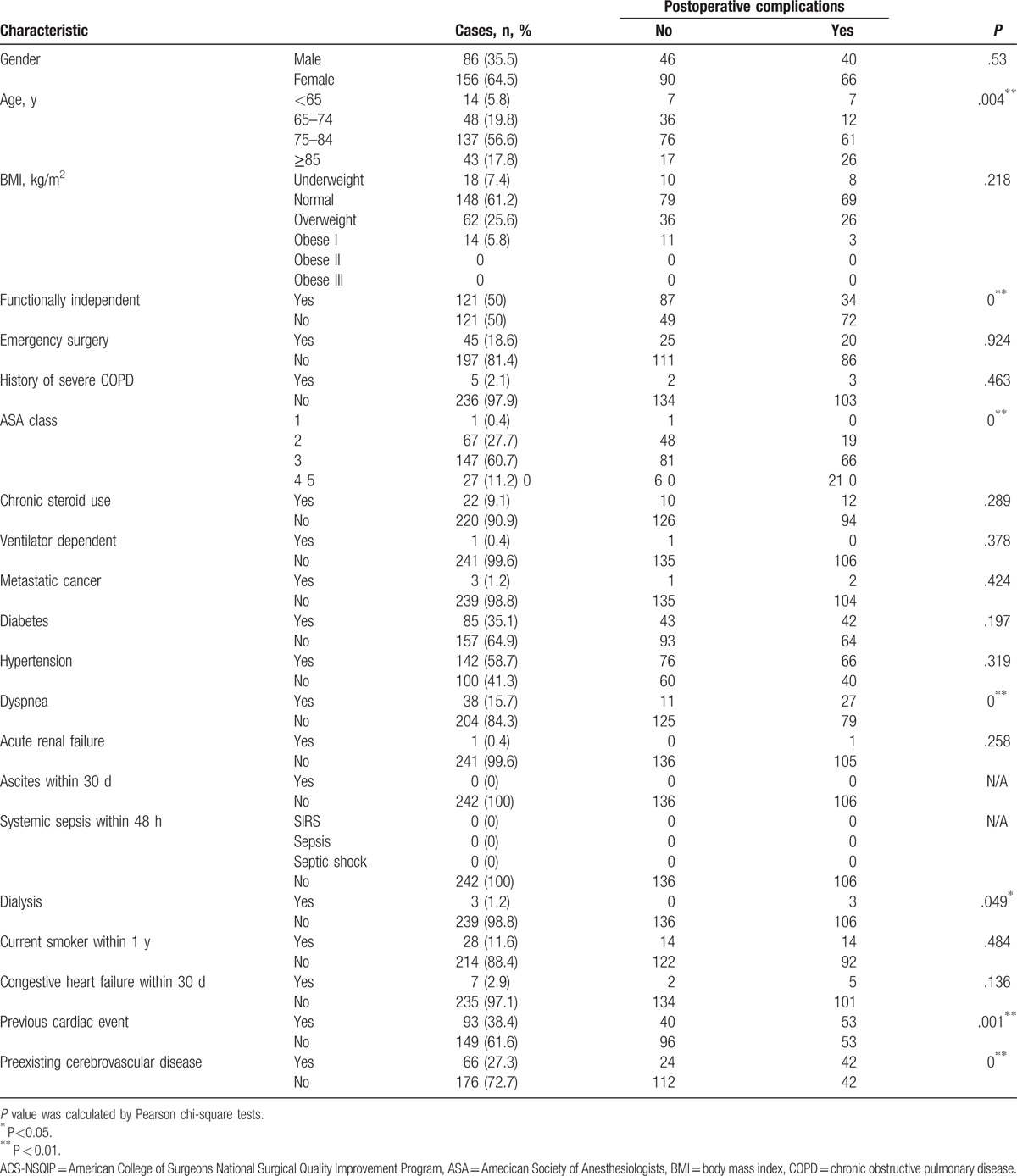
Postoperative complications in the study population (N = 242) predicted by the ACS-NSQIP calculator based on patient characteristics.

Additional risk factors, including preexisting cardiac event and cerebrovascular disease, are presented in Table 2. In this study population, 93 patients (38.4%) had a prior cardiac event including coronary heart disease, arrhythmia, and myocardiosis; 66 patients (27.3%) had cerebrovascular disease. Thus, postoperative complications were significantly more prevalent in patients with a history of cerebrovascular disease (*P* = 0; Table 2) and preexisting cardiac event (*P* = .001; Table 2).

### 3.2 Predicted risk of postoperative complications

**Figure 2:**
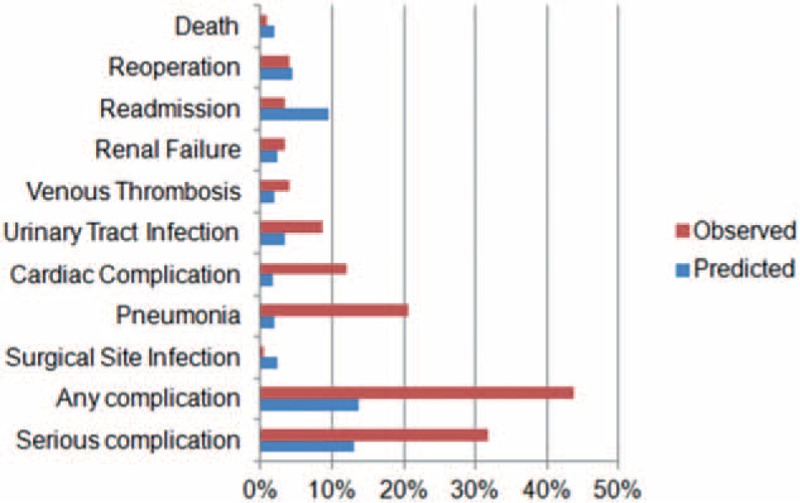
Observed and predicted postoperative complications in the study population (N = 242); observed prevalence: red; predicted prevalence: blue.

The ACS-NSQIP risk calculator was used for predicting postoperative risk (Fig. 2). Observed and predicted incidence of all postoperative complications was 43.8% and 13.7% (±5.9%) (*P* < .01), while that of serious postoperative complications was 31.8% and 13.0% (±5.6%) (*P* < .01), respectively. Differences between predicted and observed prevalence were found for all postoperative complications, such as cardiac complication (predicted 1.6% [±1.5%] vs observed 12.0%; *P* < .001) and hospital stay (predicted 5.7 [±1.9] days vs observed 19.9 [±13.4] days; *P* < .001). Moreover, the ACS-NSQIP underestimated the rates of renal failure, urinary tract infection, deep venous thrombosis, pneumonia, and cardiac complication, whereas it overestimated the rates of surgical-site infection, reoperation, readmission, and mortality (Fig. 2).

**Figure 3:**
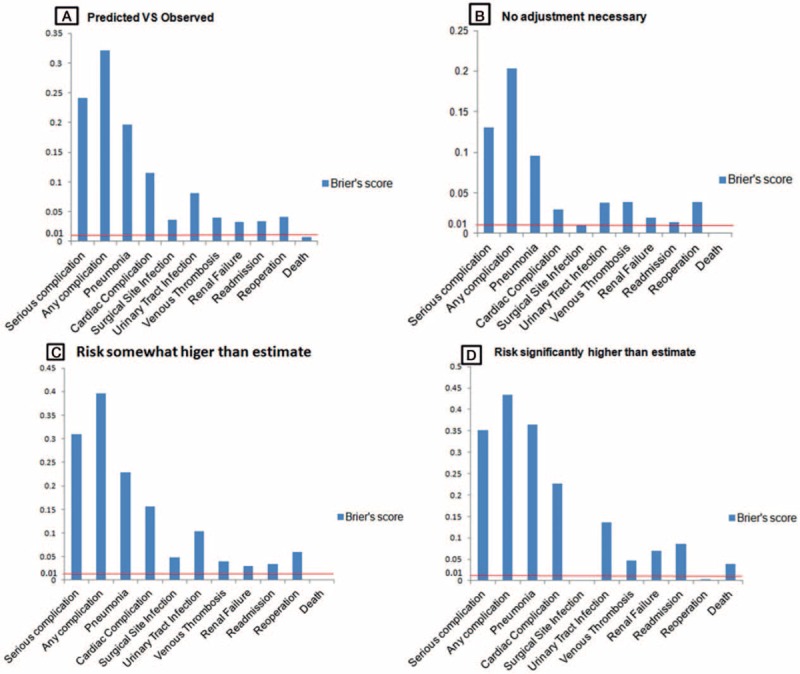
(A) Brier score of predicted cases against observed cases; Brier score of predicted cases against observed cases in different risk categories; (B) risk categorized as “No adjustment necessary”; (C) risk categorized as “Risk somewhat higher than estimated”; and (D) risk categorized as “Risk significantly higher than estimated”; red line at Brier score of 0.01 indicated good predictive accuracy.

The Hosmer–Lemeshow test demonstrated that the ACS-NSQIP model had moderate accuracy in predicting all postoperative complications (Table 3). Furthermore, B scores were calculated for evaluating predictive validity (Fig. 3A), and the ACS-NSQIP model could not accurately predict the risk of all postoperative complications (B = 0.321) or serious postoperative complications (B = 0.241). However, the ACS-NSQIP model accurately predicted the risk of death (B = 0.0072).

**Table 3:**
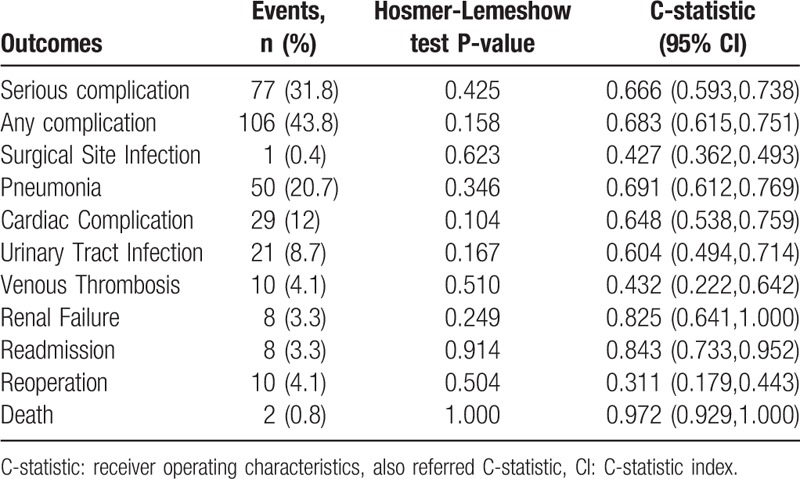
Event occurrence rates, Hosmer-Lemeshow test values, and C-statistic analysis results for postoperative complications.

**Figure 4:**
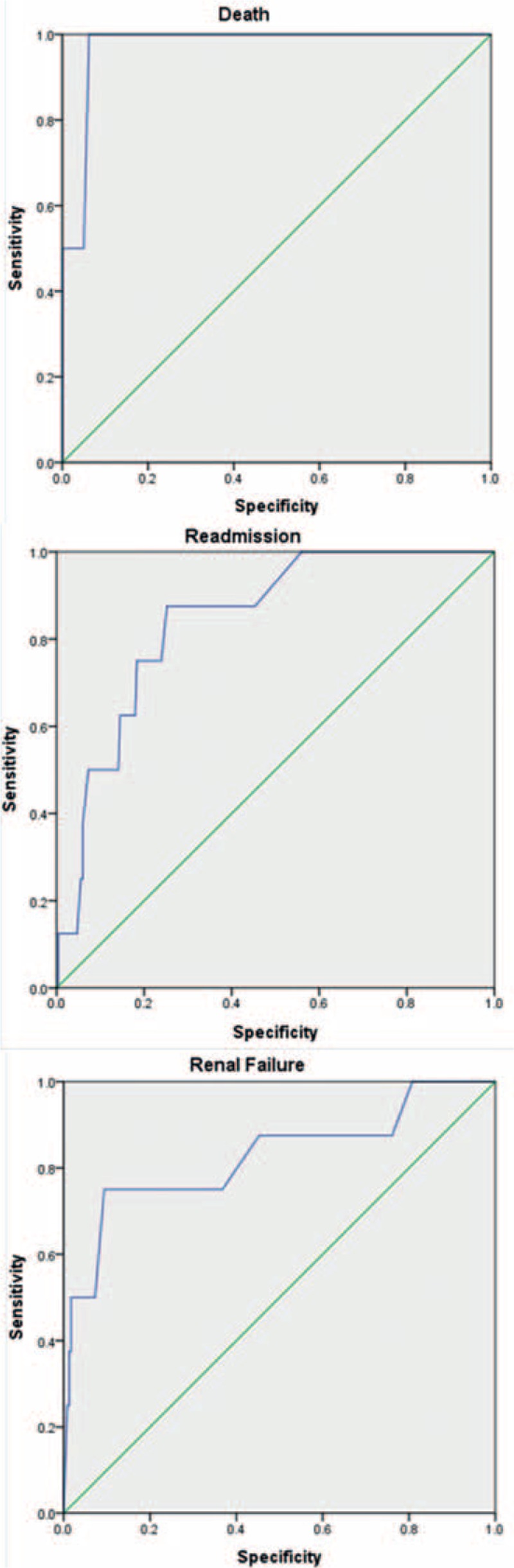
The area under the receiver operating characteristics (ROC) for renal failure, readmission and mortality within 30 days postoperation.

The ability of the ACS-NSQIP model to predict postoperative complications of lumbar surgery was evaluated using receiver-operating characteristics curves and calculated through areas under the curve. The areas under the curve for predicting any complications, serious complications, renal failure, readmission, or death were 0.683 (95% C-statistic index [CI] 0.615–0.751), 0.666 (95% CI 0.593–0.783), 0.825 (95% CI 0.641–1.00), 0.843 (95% CI 0.733–0.952), and 0.972 (95% CI 0.929–1.00), respectively (Table 3, Fig. 4). We further evaluated the predictive accuracy of the ACS-NSQIP model in 3 categories of surgeon-adjusted risks (Fig. 3): there were 102 (42.1%) patients in the “no adjustment necessary”, 99 (40.1%) in the “risk somewhat higher than estimated”, and 41 (16.9%) in the “risk significantly higher than estimated” groups. In patients with no adjustment, the B score showed that the ACS-NSQIP model was accurate in predicting risk of postoperative surgical site infection and death (Fig. 3B). Further, the ACS-NSQIP model accurately predicted risk of death among patients with risk somewhat higher than estimated (Fig. 3C), and risk of surgical-site infection and reoperation among patients with risk significantly higher than estimated (Fig. 3D).

## 4 Discussion

In this study, we evaluated the predictive utility of the ACS-NSQIP surgical risk calculator for postoperative complications following lumbar surgeries in elderly Chinese patients. In general, the ACS-NSQIP was not useful in predicting any or serious complications in patients undergoing lumbar surgery. It may have moderate accuracy in predicting death; however, its predictive ability in other specific compilations was impaired. Additional risk factors using surgeon-adjusted risks did not necessarily increase the accuracy of the ACS-NSQIP risk calculator.

To the best of our knowledge, this is the first study to evaluate the predictive value of the ACS-NSQIP risk calculator in patients undergoing lumbar surgery. Several studies on the utility of the ACS-NSQIP calculator have obtained various results. Dahlke et al^[9]^ reported good predictive accuracy of the ACS-NSQIP calculator in patients undergoing general and colon surgery. Similarly, Mogal et al^[10]^ showed that the ACS-NSQIP calculator had good accuracy in predicting outcomes after pancreaticoduodenectomy, in spite of a slight variation between diagnostic groups. However, more recent studies have found limited predictive value of the ASC-NSQIP in patients undergoing other types of surgeries. For example, the ACS-NSQIP calculator could not accurately reflect the risk of a subgroup of patients with more serious complications after laparoscopic colectomy.^[11]^ Szender et al^[7]^ reported limitations of the ACS-NSQIP in evaluating complications in patients in gynecologic oncology. Moreover, the ACS-NSQIP model did not have utility in predicting complications of knee and hip arthroplasties,^[12]^ total laryngectomy,^[13]^ and soft-tissue sarcoma resection.^[14]^ These data, together with our results, suggest the limited value of the ACS-NSQIP risk calculator across patients undergoing different surgeries.

One reason for the limited value of the ACS-NSQIP calculator in this study could be attributed to patient characteristics. Our cohort comprised of patients older than 60 years of age and, therefore, may have potentially been at a higher risk for organ deficiency that, in turn, increased their risk of postoperative complications. In this study, we added risk factors that additionally contributed to the risk of surgical complications in order to increase the accuracy of the ASC-NSQIP model. However, the results were not satisfactory. In patients with “risk somewhat higher than estimated” and “risk significantly higher than estimated”, the ASC-NSQIP model could not discriminate all or serious complications, which indicates that important factors were missed in the composition of this model.

Our study had potential limitations. First, the retrospective study design hampered information collection; therefore, some important factors may have been ignored in the present study. The additional surgeon-adjusted risk based on retrospective design may have introduced an element of bias in the study design. Moreover, the sample size was relatively small and, therefore, the predictive value of ASC-NSQIP model for specific complications might not be acceptable. The reoperation rate was relatively higher in our study (10%) compared to others.^[15]^ The potential reason may due to our longer hospital stay (19.9 ± 13.4 days).

## 5 Conclusion

The ACS-NSQIP surgical risk calculator is not an accurate tool for predicting postoperative complications in geriatric Chinese patients undergoing lumbar surgery. Further studies with a larger sample size and prospective design are warranted for investigating a better adjustment regimen or for developing a new risk model for the evaluation of complications in patients undergoing lumbar surgery.
